# Evaluation of Antibody Responses in Patients with B-Cell Malignancies after Two and Three Doses of Anti-SARS-CoV-2 S Vaccination—A Retrospective Cohort Study

**DOI:** 10.3390/cancers15020524

**Published:** 2023-01-14

**Authors:** Stella Rosa Maria Wirth, Klaus Podar, Martin Pecherstorfer, Philipp Wohlfarth, Ulrich Jaeger, Josef Singer

**Affiliations:** 1 Karl Landsteiner University of Health Sciences, Dr. Karl-Dorrek-Straße 30, 3500 Krems, Austria; 2 Division of Molecular Oncology and Hematology, Karl Landsteiner University of Health Sciences, Dr. Karl-Dorrek-Straße 30, 3500 Krems, Austria; 3 Department of Internal Medicine II, University Hospital Krems, Mitterweg 10, 3500 Krems, Austria; 4 Department of Internal Medicine I, Hematopoietic Stem Cell Transplantation Unit, Medical University of Vienna, 1090 Vienna, Austria; 5 Department of Internal Medicine I, Division of Hematology and Hemostaseology, Medical University of Vienna, 1090 Vienna, Austria

**Keywords:** SARS-CoV-2, COVID-19, SARS-CoV-2 S vaccine, antibody response, seroconversion, B-cell malignancies, third vaccination

## Abstract

**Simple Summary:**

Patients with B-cell malignancies benefit from the third vaccination against SARS-CoV-2 with regards to antibody production. In this cohort, patients with B-cell malignancies experienced greater difficulties in developing neutralizing antibodies, particularly under active treatment with anti-CD19 CAR T-cells or anti-CD20 monoclonal antibodies. Nevertheless, the third dose achieved an enhancement of the serological responses and increased humoral immunity. These observations are in line with recent studies. Future studies with larger cohorts on this research topic will broaden the understanding of an effective anti-SARS-CoV-2 vaccination scheme and guide the timing for upcoming booster vaccinations in high-risk hematological patients. Additionally, future studies should also include data on circulating B- and T-cells in order to gain a more complete picture of the immune system of patients with B-cell malignancies upon different treatments and their influence on immune protection in response to vaccinations.

**Abstract:**

Patients with B-cell malignancies are at a higher risk of severe SARS-CoV-2 infections. Nevertheless, extensive data on the immune responses of hematological patients and the efficacy of the third dose of the vaccine are scarce. The goal of this study was to determine standardized anti-SARS-CoV-2 S antibody levels and to evaluate differences between treatment modalities in response to the second and third vaccines among patients with B-cell malignancies treated at the University Hospital Krems and the University Hospital of Vienna. The antibody levels of a total of 80 patients were retrospectively analyzed. The results indicate a significant increase in antibody production in response to the third vaccination. The highest increases could be observed in patients in a “watchful-waiting” and “off-therapy” setting. Encouragingly, approximately one-third of patients who did not develop antibodies in response to two vaccinations achieved seroconversion after the third vaccination. “Watchful-waiting”, “off-therapy” and treatment with BTK inhibitors were indicative for increased antibody response after the third dose compared to anti-CD19 CAR T-cell and anti-CD-20 antibody treatment. In summary, the results of this study underline the pre-eminent role of the need for complete vaccination with three doses for the development of protective immunity in patients with B-cell malignancies.

## 1. Introduction

Hemato-oncological patients belong to a risk group of the COVID-19 pandemic and have a higher probability of undergoing COVID-19-induced complications [1]. Vaccines have been the primary strategy in the battle against SARS-CoV-2 [2]. They normally lead to significant humoral immune reactions, which offer protection from developing a severe acute respiratory syndrome. Patients with B-cell malignancies, however, are at risk of failing to produce the same immunological response compared to nonimmune-compromised vaccinated individuals, making them more susceptible to infection and a serious course of disease [3,4,5]. This risk is even greater in patients undergoing active immunosuppressive therapy [6]. The extent to which therapeutic measures affect vaccination against SARS-CoV-2 and the implications this poses to patients is currently the subject of intensive research. Still, data on the efficacy of the third dose of vaccination in patients with B-cell malignancies are limited and requires further research, particularly in light of the decreasing immunogenicity and evolving novel virus variants [7].

Thus, this study included a population of 80 patients with B-cell malignancies under different treatment modalities to investigate this matter further and provide more data for affected patients.

## 2. Materials and Methods

### 2.1. Patients

This retrospective study analyzed patients with B-cell malignancies (*n* = 80) at two oncologic centers: University Hospital Krems and Medical University of Vienna. The patients were analyzed according to their anti-SARS-CoV-2 S antibodies after vaccination against SARS-CoV-2. Data were taken from electronic patients’ records of routine medical visits from 1 January 2020 to 31 March 2022. 

The patients were diagnosed with Burkitt’s lymphoma (*n* = 1), chronic lymphocytic leukemia/small lymphocytic lymphoma (*n* = 30), diffuse large B-cell lymphoma (*n* = 19), follicular lymphoma (*n* = 14), hairy cell leukemia (*n* = 2), marginal zone lymphoma (*n* = 8), mantle cell lymphoma (*n* = 4) and lymphoplasmocytic lymphoma (*n* = 2). The treatments comprised anti-CD20 monoclonal antibodies (*n* = 18), anti-CD19 CAR T-cell therapy (*n* = 12), BTKi (*n* = 9), monitoring after care (“off-therapy”, *n* = 27) or in a “watchful-waiting” setting (*n* = 14). The patients in “off-therapy” underwent treatment and were under follow-up care at their respective oncologic center. The “watchful-waiting” patients did not initiate treatment and were monitored regularly at our departments, as for some of the included diseases, the patients did not require treatment at the time of diagnosis, because there is no longstanding benefit of the initiation of treatment in asymptomatic patients. Here, treatment should only be commenced upon symptomatic changes indicating disease progression.

Patients under the age of 18 were exclude, as were also patients after the application of the therapeutic antibodies sotrovimab (Xevudy^®^, GlaxoSmithKline) and tixagevimab/cilgavimab (Evusheld^®^, AstraZeneca), as these antibodies interfere with the test system.

This study was approved by the Institutional Review Board and the Ethics Committee of Lower Austria (EK No. GS4-EK-4/696-2021), as well as the Institutional Review Board and the Ethics Committee of the Medical University of Vienna (EK No. 1230/2022), and was conducted according to the Declaration of Helsinki.

### 2.2. Determination of Antibody Levels

The serum of the included patients was assessed for anti-SARS-CoV-2 S antibody levels using the Elecsys^®^ Anti-SARS-CoV-2 S test system (Roche Diagnostics, Vienna, Austria) and are displayed in binding antibody units/milliliter (BAU/mL). As the linear range of this quantitative immunoassay is from 0.4 to 250 BAU/mL, negative results are depicted as 0.39 BAU/mL. Similarly, highly reactive samples above the linear range are capped with a value of 250.01 BAU/mL in this study. Titers > 0.8 BAU/mL are considered reactive in this test system (Elecsys^®^ Anti-SARS-CoV-2 S. Package Insert 2020-09, V1.0; Material Numbers 09289267190 and 09289275190; Roche Diagnostics, Vienna, Austria).

### 2.3. Statistical Analyses

The descriptive statistics of this study included sex, age, vaccination scheme (*homologous* or *heterologous*) and oncological treatment of the patients (“watchful-waiting”, anti-CD20 treatment, anti-CD19 CAR T-cell therapy, BTKi and “off-therapy”). 

Assessment of the normal distribution of the continuous variables was performed using a Q-Q plot. The Kruskal–Wallis test was applied for multiple group comparisons, followed by Dunn’s multiple comparison test. A multivariate linear regression analysis was performed in order to identify the factors associated with antibody responses. The mean differences between two variables were evaluated with paired *t*-tests. The adequacy of the models was assessed using the R-squared and ANOVA tests. Multicollinearity was assessed using variance inflation factors to confirm the independence of the variables included in the regression model. The significance level was determined as *p* < 0.05 (two-tailed). 

* *p* < 0.05, ** *p* < 0.01 and *** *p* < 0.001

The software for the statistical analysis and graphing comprised R software, IBM SPSS Statistics for Windows v26 (IBM Corp., Armonk, NY, USA) and GraphPad Prism 9.1.2 (GraphPad Software, San Diego, CA, USA).

## 3. Results

### 3.1. Patient Population

Eighty patients were included in the study (37 female, 43 male; median age: 71 years, range: 40−98 years). Sixty-eight patients were treated at the Department for Internal Medicine 2, University Krems, and twelve patients received CAR T-cell therapy at the Medical University of Vienna. The age span between patient groups was 40 to 98 years, with a median overall age of 71 years. There was a slight male predominance (54% male, 46% female) in the patient population.

The patients were divided into five groups depending on the treatment: 13 out of 80 patients were monitored in a “watchful-waiting” setting (6 female, 7 male; median age: 74 years, range: 61−84 years), 19 patients were treated with anti-CD20 therapy (8 female, 11 male; median age: 71 years, range: 40−98 years), 12 patients received anti-CD19 CAR T-cell therapy (6 female, 6 male; median age: 61.5 years, range: 43−79 years), 9 patients were under BTK-inhibition (3 female, 6 male; median age: 68 years, range: 55−81 years) and 27 patients belonged to the “off-therapy” group (14 female, 13 male; median age: 72.5 years, range: 46−89 years). 

Except for one patient, who did not receive the third vaccination due to the fact of a past COVID-19 infection, all patients were vaccinated three times at the end of the assessment period (see Table 1). Thirty-nine patients received the same vaccination three times (*homologous* vaccination scheme), and 40 patients were supplied with a *heterologous* vaccination scheme.

The patients received their second dose between February and December 2021 and their third dose between July 2021 and January 2022. The time interval between the second and third vaccination spanned from 3 to 9 months. 

### 3.2. Anti-SARS-CoV-2 S Antibody Levels in Patients with B-Cell Malignancies

The presence of anti-SARS-CoV-2 S antibodies was determined during routine check-ups at the two oncologic centers. The titer values assessed after the second and third vaccination were analyzed. Titers reaching levels >0.8 BAU/mL were considered to be reactive. 

**Figure 1:**
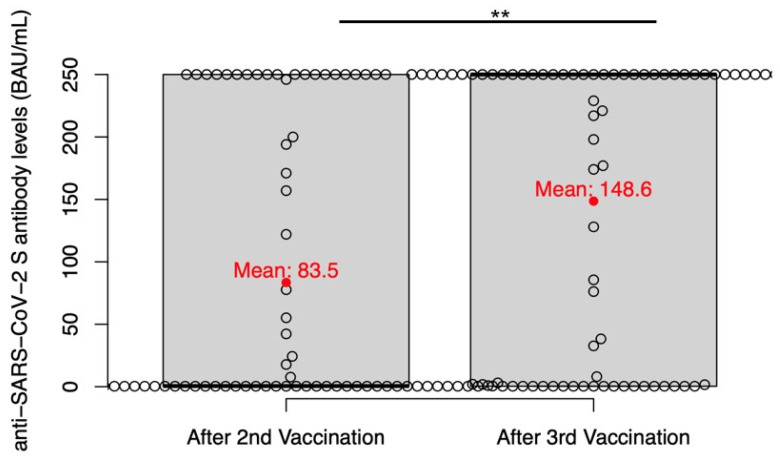
Comparison of anti-SARS-CoV-2 antibody levels after the 2nd and 3rd vaccination. The dots illustrate the antibody titer levels of individual cases. The median values are displayed with bold lines. The mean values are displayed as red dots. BAU/mL: binding antibody units/mL. ** *p* < 0.01.

Overall, after the second vaccination, half of the patients showed no antibody production (depicted as 0.39 BAU/mL), and 26.32% produced antibody levels above the linear range of the immunoassay (depicted as 250.01 BAU/mL). After two vaccinations, the median antibody titer was 0.54 BAU/mL and the mean was 83.5 BAU/mL (Figure 1). 

After three vaccinations, an increased proportion of patients (50.68%) produced neutralizing antibodies above the linear range of the test (depicted as 250.01 BAU/mL). On the contrary, approximately a quarter of the patients (27.40%) still failed to produce reactive antibodies. Thus, after three vaccinations patients displayed median BAU/mL values of 250.0 BAU/mL. The mean was 148.6 BAU/mL, which was a statistically significant increase compared to after the second vaccination (*p* = 0.001).

### 3.3. Seroconversion according to Treatment Modality 

**Figure 2:**
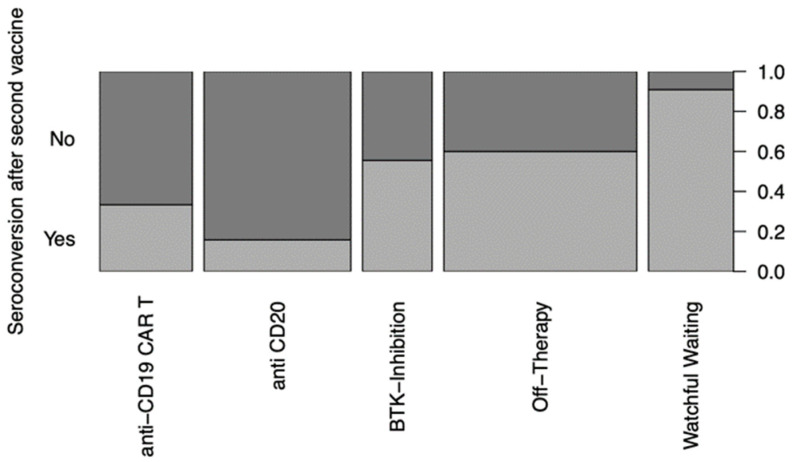
Mosaic plot of seroconversion with a >0.8 BAU/mL after the 2nd vaccination distributed by treatment modality. Anti-CD19 CAR T (*n* = 12), anti-CD20 treatment (*n* = 19), BTKi (*n* = 9), “off-therapy” (*n* = 25) and “watchful waiting” (*n* = 10).

**Figure 3:**
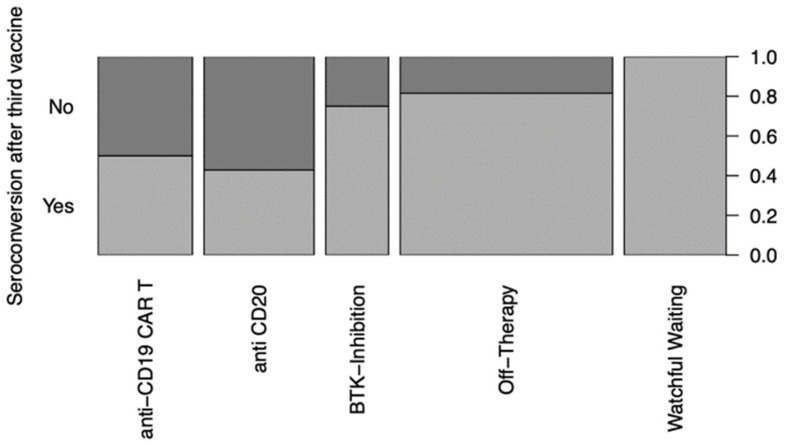
Mosaic plot of seroconversion with a >0.8 BAU/mL after the 3rd vaccination distributed by treatment modality. Anti-CD19 CAR T (*n* = 12), anti-CD20 treatment (*n* = 14), BTKi (*n* = 8), “off-therapy” (*n* = 27) and “watchful waiting” (*n* = 13).

In the “watchful-waiting” cohort, 9 patients managed seroconversion with a BAU/mL > 0.8 after the second vaccination (Figure 2); after the third dose, all 13 patients achieved seroconversion (Figure 3). While 3 out of 19 patients receiving anti-CD20 therapy managed to develop reactive antibodies (BAU/mL > 0.8) after the second vaccination (Figure 2), 6 patients did so after the third vaccination (Figure 3). After the second vaccination, four of the twelve patients receiving CAR T-cell therapy achieved seroconversion (Figure 2). This number increased to six after the third vaccination (Figure 3). Five patients treated with BTKi developed neutralizing antibodies in response to the second vaccination (Figure 2) and six after the third (Figure 3). After the second vaccination, 15 patients in the “off-therapy” cohort developed neutralizing antibodies (Figure 2); after the third, this number increased to 22 (Figure 3). In summary, certain differences in seroconversion between the therapy groups could be observed, with the patients receiving anti-CD19 CAR T-cell and anti-CD20 therapy having greater difficulties producing antibodies in response to the vaccinations than the patients in the “off-therapy” and “watchful-waiting” cohorts.

### 3.4. Impact of Oncologic Treatment on Anti-SARS-CoV-2 S Vaccine Immune Responses

**Figure 4:**
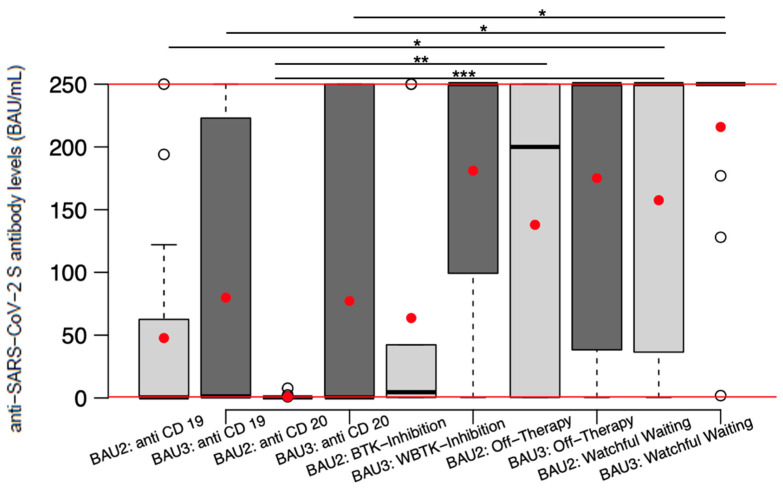
Comparison of the antibody levels induced after the 2nd and 3rd vaccinations by treatment modality. The boxplots indicate the minimum score, lower quartile, median (bold line), mean (red dot), upper quartile and maximum score. The shaded, gray boxes illustrate the interquartile range (middle 50% of data). The whiskers (dashed line) indicate the antibody levels within 1.5× of the interquartile range from the quartiles (nearest the box edge). The outliers (white circles) are outside 1.5× of the interquartile range, above or below the upper and lower quartiles. The threshold (lower red line) indicates seroconversion at >0.8 BAU/mL; the upper red line indicates the upper limit of the linear range of the assay (250 BAU/mL). BAU2: antibody values after 2 vaccinations; BAU3: antibody values after 3 vaccinations * *p* < 0.05, ** *p* < 0.01 and *** *p* < 0.001.

As can be seen in Figure 4, among all treatment modalities, patients in the aftercare (“off-therapy”) and in the “watchful-waiting” cohorts developed the highest antibody levels in response to the second and third vaccinations. After both the second and third vaccinations, the “watchful-waiting” group displayed median antibody levels coinciding with the maximum of the titer range (median_vacc2_ 95% CI of median: 17.8–250.0 BAU/mL; median_vacc3_ 95% CI of median: 250.0–250.0 BAU/mL). The subgroup comparison of the treatment modalities showed that the patients in the “watchful-waiting” setting produced significantly higher antibody titers compared to the patients treated with anti-CD20 monoclonal antibodies and anti-CD19 CAR T-cell therapy after both vaccination timepoints (anti-CD20_vacc2_ *p* = 0.000, anti-CD19_vacc2_ *p* = 0.045; anti-CD20_vacc3_ *p* = 0.018, anti-CD19_vacc3_ *p* = 0.017). The patients in the “off-therapy” group had an increase of median antibody values from 200 to 250.0 BAU/mL between the second and third vaccines. The “off-therapy” patients also developed significantly higher antibody responses than patients receiving anti-CD20 treatment. The anti-CD20-treated patients had median antibody levels <0.8 BAU/mL after both vaccination timepoints (median_vacc2_ 0.39 BAU/mL; 95% CI of median: 0.39–0.39 BAU/mL; median_vacc3_: 0.39 BAU/mL; 95% CI of median: 0.39–250.0 BAU/mL). Our data show that patients treated with anti-CD20 monoclonal antibodies had significantly greater difficulties producing high antibody titers in response to the second vaccine compared to both the “watchful-waiting” and “off-therapy” patients (W-W_vacc2_ *p* = 0.000, off-therapy_vacc2_ *p* = 0.045). However, due to the increase in antibody production, the patients receiving anti-CD20 therapy had a reduced antibody response compared to patients in “watchful-waiting” but not to patients in “off-therapy” after the third vaccine (W-W_vacc3_ *p* = 0.018). The patients treated with anti-CD19 CAR T-cell therapy displayed median antibody levels of 0.39 BAU/mL after the second vaccination. These patients, however, managed to achieve median antibody levels above the seroconversion threshold of >0.8 BAU/mL after the third dose (CAR T-cell therapy median_vacc2_: 0.39 BAU/mL; 95% CI of median: 0.39.0–62.55 BAU/mL; CAR T-cell therapy median_vacc3_: 1.22 BAU/mL; 95% CI of median: 0.39–229.0 BAU/mL). Nonetheless, patients undergoing CAR T-cell treatment showed significantly less antibody production compared to the patients in the “watchful-waiting” cohort after the second and third vaccinations (compared to W-W_vacc2_ *p* = 0.045, compared to W-W_vacc3_ *p* = 0.017). The patients receiving BTKi managed the largest increase in median antibody production between the two vaccinations, increasing from 4.58 BAU/mL to the maximum 250.0 BAU/mL, but they did not show significant differences in vaccine-induced anti-SARS-COV-2 S antibodies in comparison to the other treatment modalities (Figure 4).

**Figure 5:**
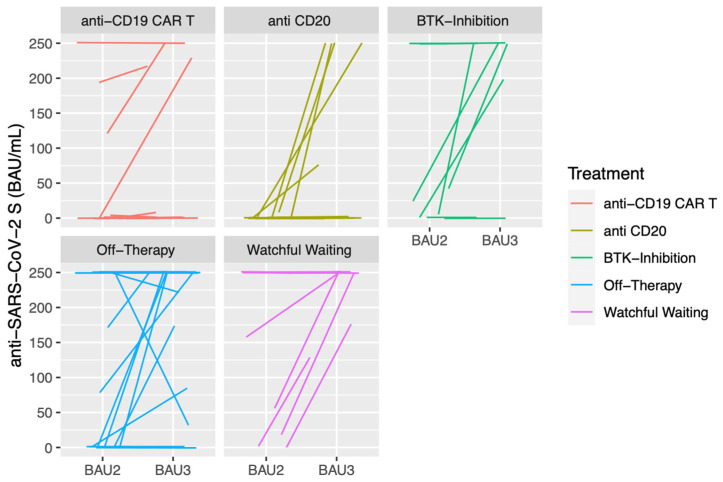
Antibody kinetics of the maximal anti-SARS-CoV-2 S antibody levels in BAU/mL after the 2nd (BAU2) and 3rd (BAU3) vaccinations by treatment modality.

Finally, the antibody kinetics of the maximal anti-SARS-CoV-2 S antibody levels in BAU/mL after the 2nd and 3rd vaccination in detail for each patient and treatment modality are displayed (Figure 5).

### 3.5. Multivariate Analysis of Factors Influencing Anti-SARS-CoV-2 S Vaccine-Induced Antibody Responses

Multivariate regression analysis was performed to identify whether the factors sex, age, vaccination scheme (*homologous* or *heterologous*) and ongoing oncological treatment (“watchful-waiting”, anti-CD20 treatment, anti-CD19 CAR T-cell therapy, BTKi and “off-therapy”) were predictors for anti-SARS-CoV-2 S antibody levels, as well as seroconversion. 

Management with “watchful-waiting”, “off-therapy” and younger age were highly significant determinants for higher antibody responses after the second vaccination (see Table 2). Correspondingly, “watchful-waiting” and “off-therapy” were indicative for increased vaccine-induced anti-SARS-COV-2 S antibodies after the third dose. Here, treatment with BTKi was also a significant predictor for higher antibody response after the third vaccination. In this cohort, age did not have a significant impact. “Watchful-waiting” was a strong determinant of achieving seroconversion in response to the second vaccination. Similarly, after the third dose of the vaccine, the modality with the addition of “off-therapy” observation were significant indicators for seroconversion.

## 4. Discussion

Vaccination against SARS-CoV-2 is a promising measure to reduce severe disease, hospitalization and mortality in the course of an infection with SARS-CoV-2. This also includes booster doses that are crucial for sustained protection. Although public health strategies are increasingly focusing on the identification and protection of vulnerable groups, extensive data on the immune responses of oncological patients are still scarce. The increased risk of a symptomatic SARS-CoV-2 infection and severe complications in patients with B-cell malignancies have been observed since the beginning of the pandemic. Studies show that the hematological patients have greater difficulties developing immunogenicity and have a higher probability of serologic nonresponse after vaccination compared to the public [8,9,10,11].

The evidence indicates a reduced development of neutralizing antibodies against SARS-CoV-2 in patients with non-Hodgkin’s lymphoma undergoing active treatment, predominantly with anti-CD20 monoclonal antibodies, Bruton’s tyrosine kinase inhibitors and the Bcl2-inhibitor Venetoclax [6,12]. Data on the efficacy of the third dose of the vaccine in patients with B-cell malignancies are limited, and this requires further research, particularly in light of the decreasing immunogenicity and evolving novel virus variants [7].

Thus, this study retrospectively analyzed the anti-SARS-CoV-2 S titers of 80 patients with B-cell malignancies, treated at the University Hospital Krems and the Medical University of Vienna between 1 January 2020 and 31 March 2022. The patients received the following vaccines: the mRNA-based vaccines BNT162b2 (Comirnaty^®^, Pfizer, Inc., New York, NY, USA) and mRNA-1273 (COVID-19 Vaccine Moderna^®^, ModernaTX Inc., Cambridge, MA, USA), as well as the vector-based vaccines AZD1222 (ChAdOx1, Vaxzevria^®^, AstraZeneca, Cambridge, UK) and JNJ-78436735 (Ad26.COV2.S, COVID-19 Vaccine Janssen^®^, Janssen, Beerse, Belgium). The vaccinations were administered either by the nationwide vaccination program of the Federal Republic of Austria or by the in-house vaccination programs at the respective hospitals. In line with being the first EMA-approved vaccine, the mRNA-vaccine Comirnaty^®^ was predominantly applied in our study cohort. The majority of patients received their third vaccine between autumn and winter of 2022. Due to the fact of its relatively late EMA approval, in March 2021, only one patient received the COVID-19 vaccine Janssen^®^. Our results indicate a significant increase in antibody production in response to the third vaccination. These data are in line with recently published studies that show a sustainable increase in antibody responses following the third vaccination [6,7,12]. Encouraging is the observation that approximately 31% (12/39) of patients who did not develop antibodies in response to two vaccinations were able to achieve seroconversion after the third vaccination. Our data on third dose efficacy are similar to recent studies reporting approximately a quarter to one-third of patients with B-cell malignancies managing seroconversion after the third vaccination, who previously had responded weakly after dose two [7,12,13].

After the second and third vaccinations, our data show that patients who were “off-therapy” or in a “watchful-waiting” setting developed the highest antibody levels. The “watchful-waiting” cohort managed to produce higher antibody titers than patients treated with both anti-CD20 monoclonal antibodies and anti-CD19 CAR T-cell therapy. It is also noteworthy to mention that several other studies, which were recently published during the finalization and review period of this paper, indicate that active treatment with anti-CD20 monoclonal antibodies is an important prognostic factor for a poor antibody response after vaccination [12,13,14,15,16,17,18,19,20,21,22].

The aim of this study was not to compare different vaccines; however, it was analyzed whether sex, age, vaccination schemes (*homologous* or *heterologous*) and ongoing oncological treatment were predictors for anti-SARS-CoV-2 S antibody levels, as well as seroconversion (Table 2). The analysis illustrates that “watchful-waiting”, “off-therapy” and younger age were highly significant determinants for higher antibody responses after the second vaccine. Treatment with BTK inhibitors was indicative for increased vaccine-induced-SARS-COV-2 S antibodies only after the third vaccine. Age was only a determinant factor for immune responses after the second vaccine. For gender differences and vaccination scheme, neither seemed to influence the immune response or seroconversion after the second or third vaccine in our patient cohort. “Watchful-waiting” was a strong predictor for seroconversion in response to the second and third doses. In addition, after the third dose, “off-therapy” observation was a significant indicator for seroconversion.

Patients that did not develop seroconversion after three vaccinations should be treated with special emphasis in future studies. Here, a fourth vaccination could be beneficial, as Benjamini et al. very recently demonstrated in a population of CLL patients that although seroconversion rates only slightly increased after a 4th vaccination with BNT162b2 (from 49.3% to 55.2%), T-cell responses improved from 42.3% of patients after the 3rd vaccination to 84.6% after 2 weeks following a 4th vaccination [23], thus indicating a beneficial effect of a second booster vaccination.

### Limitations

A limitation of this study was the low number of included patients due to the restricted time span of the data collection between January 2020 and March 2022. Furthermore, several eligible patients could not be included due to the fact of missing data, application of therapeutic antibodies or death within the observation period. Consequently, low numbers of patients are represented in the “watchful-waiting”, anti-CD19 CAR T-cell and BTK inhibitor cohort, leading to an uneven distribution within the treatment groups and the necessity for future studies with larger sample sizes. An additional limitation was that the maximal titer values exceeding the linear range of the Elecsys^®^ Anti-SARS-CoV-2 S test could not be analyzed. These values would provide more comprehensive insight into the distinction between patient groups and the development of antibody kinetics over time. Similarly, the evaluation of the effective timepoints for booster vaccinations in vulnerable patient groups would be possible. Thus, the indication for future studies to determine the exact values of BAU/mL is given. 

Moreover, circulating immune cells were not analyzed in this study. It would be especially interesting for the anti-CD19- and anti-CD20-treated patients to determine the amount of circulating B-cells and the amount of circulating anti-CD19 CAR T cells. Unfortunately, these parameters were determined only in certain cases independently of the second and third vaccinations and could thus not be analyzed. 

Additionally, the evaluation of the presence and quantity of T-cell responses against SARS-CoV-2 vaccinations could clarify the role of the protection of cellular immunity in patients with impaired or masked humoral immunity. Therefore, more research should be conducted on the sustainability of cellular immune responses. 

## 5. Conclusions

Whether complete vaccination with three doses is imperative for immune protection from COVID-19 disease in the immunocompetent population, and hemato-oncologic patients in particular, is yet to be evaluated. The patients in our cohorts experienced greater difficulties developing neutralizing antibodies, particularly under active treatment with anti-CD19 CAR T-cells or anti-CD20 monoclonal antibodies. Nevertheless, the third dose achieved an enhancement of the serological responses and increased humoral immunity. These observations are in line with several recent studies. It can be concluded that complete vaccination schemes with three doses, particularly considering the emergence of new SARS-CoV-2 variants and time-dependent humoral immunity, are crucial for patients with B-cell malignancies. Future studies with larger cohorts on this research topic will broaden the understanding of an effective anti-SARS-CoV-2 vaccination scheme and guide the timing for upcoming booster vaccinations in high-risk hematological patients. Additionally, future studies should also include data on circulating B- and T-cells in order to gain a more complete picture of the immune system of patients with B-cell malignancies upon different treatments and their influence on immune protection in response to vaccinations.

## Figures and Tables

**Table 1:** Frequencies and percentages of patients by vaccination received (1st, 2nd and 3rd).

Type of Vaccine	1st Vaccination		2nd Vaccination		3rd Vaccination	
	*n*	%	*n*	%	*n*	%
Vaxzevria^®^, AstraZeneca	13	16.3	13	16.3	3	3.8
Comirnaty^®^, BioNTech/Pfizer	38	47.5	38	47.5	57	71.3
COVID-19 Vaccine Moderna^®^, Moderna	29	36.3	29	36.3	18	22.5
COVID-19 Vaccine Janssen^®^, Janssen					1	1.3
Missing	-	-	-	-	1	1.3
	80	100	80	100	80	100

**Table 2:** Variables associated with the antibody response and seroconversion after the 2nd and 3rd anti-SARS-COV-2 S vaccinations in patients with B-cell malignancies (*n* = 80). The multivariate regression analysis included oncological treatment (anti-CD19 CAR T-cell therapy, anti-CD20 therapy, BTK-inhibition, “off-therapy” and “watchful-waiting”), as well as sex, age and vaccination scheme (*homologous* or *heterologous*) of the patients. The intercept serves as the reference category, if all independent variables equal 0. Significant *p*-values are displayed in bold. BTKi: Bruton’s tyrosine kinase inhibitor.

Patient Group	Factor	Unstandardized Coefficients Beta	*p*-Value	Adjusted R^2^	F-Value
**Second vaccine**	Intercept	202.700	0.007	0.29	5.5
	Anti-CD20 treatment	−30.280	0.399	-	-
	BTKi	36.081	0.401	-	-
	Off-therapy	101.252	**0.004**	-	-
	Watchful-waiting	128.047	**0.002**	-	-
	Sex	−1.129	0.961	-	-
	Age	−2.192	**0.045**	-	-
	Vaccination scheme	−37.875	0.096	-	-
**Third vaccine**	Intercept	198.281	0.022	0.17	3.1
	Anti-CD20 treatment	12.564	0.772	-	-
	BTKi	114.136	**0.025**	-	-
	Off-therapy	106.009	**0.007**	-	-
	Watchful-waiting	151.565	**0.001**	-	-
	Sex	−4.699	0.859	-	-
	Age	−1.650	0.195	-	-
	Vaccination scheme	−28.018	0.279	-	-
**Seroconversion after second vaccine**	Intercept	1.708	6.01 × 10^−6^	0.20	3.7
	Anti-CD20 treatment	−0.128	0.455	-	-
	BTKi	0.293	0.156	-	-
	Off-therapy	0.285	0.082	-	-
	Watchful-waiting	0.626	**0.002**	-	-
	Sex	0.064	0.558	-	-
	Age	−0.005	0.305	-	-
	Vaccination scheme	−0.157	0.148	-	-
**Seroconversion after third vaccine**	Intercept	1.745	1.61 × 10^−6^	0.16	2.9
	Anti-CD20 treatment	−0.038	0.823	-	-
	BTKi	0.279	0.157	-	-
	Off-therapy	0.319	**0.037**	-	-
	Watchful-waiting	0.515	**0.005**	-	-
	Sex	−0.046	0.659	-	-
	Age	−0.002	0.646	-	-
	Vaccination scheme	−0.171	0.094	-	-

## Data Availability

The data remain at the Medical University of Vienna and the University Hospital Krems. The datasets used and analyzed during the current study are available from the corresponding author upon reasonable request.
